# Constraints on the Transfer of Perceptual Learning in Accented Speech

**DOI:** 10.3389/fpsyg.2013.00148

**Published:** 2013-04-01

**Authors:** Frank Eisner, Alissa Melinger, Andrea Weber

**Affiliations:** ^1^Max Planck Institute for PsycholinguisticsNijmegen, Netherlands; ^2^School of Psychology, University of DundeeDundee, UK; ^3^Donders Institute for Brain, Cognition and Behaviour, Radboud University NijmegenNijmegen, Netherlands

**Keywords:** speech, perceptual learning, foreign-accented speech, cross-modal priming

## Abstract

The perception of speech sounds can be re-tuned through a mechanism of lexically driven perceptual learning after exposure to instances of atypical speech production. This study asked whether this re-tuning is sensitive to the position of the atypical sound within the word. We investigated perceptual learning using English voiced stop consonants, which are commonly devoiced in word-final position by Dutch learners of English. After exposure to a Dutch learner’s productions of devoiced stops in word-final position (but not in any other positions), British English (BE) listeners showed evidence of perceptual learning in a subsequent cross-modal priming task, where auditory primes with devoiced final stops (e.g., “seed”, pronounced [si:t^h^]), facilitated recognition of visual targets with voiced final stops (e.g., SEED). In Experiment 1, this learning effect generalized to test pairs where the critical contrast was in word-initial position, e.g., auditory primes such as “town” facilitated recognition of visual targets like DOWN. Control listeners, who had not heard any stops by the speaker during exposure, showed no learning effects. The generalization to word-initial position did not occur when participants had also heard correctly voiced, word-initial stops during exposure (Experiment 2), and when the speaker was a native BE speaker who mimicked the word-final devoicing (Experiment 3). The readiness of the perceptual system to generalize a previously learned adjustment to other positions within the word thus appears to be modulated by distributional properties of the speech input, as well as by the perceived sociophonetic characteristics of the speaker. The results suggest that the transfer of pre-lexical perceptual adjustments that occur through lexically driven learning can be affected by a combination of acoustic, phonological, and sociophonetic factors.

## Introduction

Laboratory experiments in spoken-language research often use highly stylized samples of speech – recorded without background noise or interference from other talkers, spoken in canonical form and free of mispronunciations, accents, changes in speaking rate, or emotional tone. Researchers aim to keep those factors as constant and controlled as possible in order to avoid sources of variance in the data they gather. There is no doubt that fluctuations in the acoustic environment as well as inter- and intra-speaker variation have an effect on perception, which is generally detrimental (e.g., Dupoux and Green, 1997; Mullennix et al., 2002; Peelle and Wingfield, 2005; Adank et al., 2009; Bent et al., 2009). However, listeners can usually learn to cope with such sources of variance, and a fairly recent body of research has sketched out the perceptual mechanisms that underlie this adaptability. This research has shown that, although sources of variability in speech cause listeners processing problems initially, these problems can often be overcome, sometimes quite rapidly, through perceptual learning. Perceptual learning allows listeners to adjust to variation in speech in a variety of difficult listening situations, including spectral and temporal degradation of the signal, accents, talker variability, and talker-idiosyncratic mispronunciations (e.g., Nygaard et al., 1994; Rosen et al., 1999; Norris et al., 2003; Bradlow and Bent, 2008; Adank and Janse, 2009). This learning may be guided by a variety of cues that are present in speech, such as visual information from the face of the talker (Bertelson et al., 2003), lexical and phonotactic knowledge (Norris et al., 2003; Cutler et al., 2008), and contingencies in acoustic-phonetic cues (Idemaru and Holt, 2011).

This study investigated lexically driven perceptual learning – one particular mechanism by which pre-lexical representations of speech sounds can be rapidly adjusted (Norris et al., 2003) and which has been studied quite extensively (see Samuel and Kraljic, 2009 for a recent review). The learning takes place when listeners repeatedly encounter a speaker’s consistently atypical productions of a speech sound, and when those atypical productions are produced in a context that allows the listener to infer the sound’s identity. As the outcome of learning, the perceptual category boundary for that speech sound is adjusted. For example, in the study by Norris et al. (2003), after listeners had heard a fricative that was midway in between /s/ and /f/ in the context of words that biased the interpretation of that sound toward /f/, they shifted their /s/-/f/ category boundary toward /f/. When a different group heard that same ambiguous fricative embedded in words that biased its interpretation toward /s/, they then showed a category boundary shift toward /s/.

Perceptual learning is an essential mechanism that allows the listener to adjust to unusual or unexpected characteristics of the speech input. However, for perceptual learning to produce optimal outcomes, it must find a balance between, on the one hand, being robust and stable in the face of constant variability between tokens, and on the other hand, being flexible enough to adapt to systematic and predictable differences. How it achieves this balance is at the heart of our investigation. To maximize stability, the learning system may be very conservative, never generalizing beyond the specific situations that the listener has encountered. Several studies have found that learning can indeed be specific, for example, for a particular talker (Eisner and McQueen, 2005; Kraljic and Samuel, 2005). However, it seems that maximum stability is not a principal property of perceptual learning under all conditions, as learned adjustments can generalize beyond the characteristics of the exposure items, for example to a different place of articulation or to other words containing that sound (Kraljic and Samuel, 2006; McQueen et al., 2006; Maye et al., 2008). It is as yet unclear under what circumstances a learned adjustment will generalize or along which dimensions it will be generalized. Here, we further investigated this basic property of lexically driven perceptual learning by testing (1) whether the change in pre-lexical representation can encode information about the position of the critical sound within a word, and (2) whether learning is affected by sociophonetic characteristics of the talker.

With respect to the first question, linking phonological information with the change in category boundary might protect the perceptual system from overgeneralizing learning in cases where the category change only occurs in a specific position. Two previous studies have reported conflicting results regarding this question. Investigating cross-positional perceptual learning of ambiguous fricatives, Jesse and McQueen (2011) found full transfer of learning from word-final position to the initial position of non-sense syllables. However, Dahan and Mead (2010), using spectrally degraded speech, reported position-sensitive generalization of learning. In their study, consonants were more readily identified when they occurred in the same word position during learning and test than when they occurred in different positions. In the current study, a [d/t] stop contrast which is commonly devoiced word-finally in Dutch-accented English (Warner et al., 2004), but not in other positions, was chosen to investigate the question of position-specific learning. Learning in such a case would be beneficial for recognizing words in which the affected phoneme category occurs in that position, but might in fact hinder word recognition when applied in positions where it is not warranted. The generalizability of learning about a position-specific accent feature was therefore tested in the context of word recognition.

With respect to the second question, the readiness of listeners to adjust a category boundary, and the generalizability of that potential learning, may be affected by the listener’s expectation regarding the speaker, specifically the likelihood that the speaker produces idiosyncratic pronunciations. The perceived identity of a speaker, or their membership to an accent community, is known to affect speech perception on a pre-lexical level (Hay et al., 2006a,b; Hay and Drager, 2010). Listeners have also been shown to process syntactic errors differently when they occur in a speaker who has a global foreign accent, compared to a speaker with a native accent (Hanulíková et al., 2012). This study tested whether such a sociophonetic effect may exist in perceptual learning at the acoustic-phonetic level, by comparing listener adjustment to word-final devoicing in foreign-accented and native-accented speech.

In three experiments using an exposure-test paradigm (McQueen et al., 2006), British English (BE) listeners first learned to adjust to word-final stop consonant devoicing in the context of performing a lexical-decision task and were then tested in a cross-modal priming task to establish whether there was a benefit of exposure in recognizing a new set of word-finally devoiced words. The experiments also included a condition in which word recognition was tested with word-initially unvoiced sounds in order to test for potential generalization of learning across word positions.

## Experiment 1

### Materials and methods

#### Participants

Twenty-four undergraduate students who were enrolled at the University of Dundee participated in exchange for course credit. All participants were native speakers of English, did not speak Dutch, and reported no hearing-related disorders. Participants gave informed consent before taking part in the study.

#### Speech materials

Stimuli were made from recordings of a female native Dutch speaker who had studied English at high-school level and who had not spent time living in an English-speaking country. The speaker had a good command of English with a noticeable Dutch accent, characterized not only by word-final stop devoicing but also other typical deviations such as substitution of alveolar stops for dental fricatives, velar fricatives for velar stops, and variation in vowel quality (Flege, 1997). Word-final devoicing was produced naturally without specific instruction, and no other substitutions occurred in the critical experimental items. Word lists were read out in a sound-damped booth, recorded with 48 kHz/16-bit sampling and stored digitally for further editing using Praat (Boersma and Weenink, n.d.). The lists consisted of 252 items in total to be used in the exposure phase (32 training words consisting of three to four syllables ending in /d/ (e.g., “overload”), 32 replacement words, matched to the training items in syllabic length and average frequency in CELEX (Baayen et al., 1995) (e.g., “surgery”), 32 /d/-initial words with three to four syllables (e.g., “delivery”), 64 filler words, as well as 92 pseudowords. Except for the word-final and word-initial /d/ in the two critical conditions, there were no other voiced stops and no other alveolar stops in the words recorded for the exposure phase. However, there were some instances of the voiced and unvoiced affricates /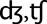/ distributed across the exposure conditions. The list for the test phase included 240 monosyllabic items (30 minimal pairs of /d/-final items (e.g., “seed”) and /t/-final items (e.g., “seat”), 30 minimal pairs of /d/-initial items (e.g., “down”) and /t/-initial items (e.g., “town”), and 120 monosyllabic filler items which did not contain stop consonants or voiced fricatives. An analysis of some of the acoustic cues that are affected by the word-final devoicing is presented in the “Acoustical analysis” section below.

#### Design and procedure

The study employed a between-subjects design in which the experimental group heard devoiced alveolar stops during an initial exposure phase, but the control group heard matched control items without stops. Both groups were then tested immediately afterward with a cross-modal priming task (following McQueen et al., 2006; Sjerps and McQueen, 2010) in which the critical conditions contrasted related vs. unrelated prime in initial vs. final position and voiced vs. voiceless alveolar stop.

During the exposure phase, participants were presented with spoken words and pseudowords and instructed to indicate after each word with a yes/no button press whether the item they had heard was an English word. The experimental and control groups both heard the 92 pseudowords and 64 filler words not containing any stop consonants. The experimental group heard in addition the 32 /d/-final items, which were substituted by the 32 matched replacement items in the control group. Three equivalent pseudo randomized orders were made for each of the two groups, and rotated across subjects. During the test phase, participants heard auditory primes paired with visual target words and pseudowords presented in succession; the task was to indicate with a yes/no button press whether the visual target was an English word. The test phase was identical for both groups.

Of the 60 words in each set of minimal pair items (/d/- and /t/-final, /d/- and /t/-initial conditions) 40 were assigned in equal proportions as prime-target pairs in a related condition (e.g., devoiced “seed” – SEED, “seat” – SEAT) and an unrelated condition (e.g., “smile” – SEED, “smile” – SEAT; visual targets are henceforth represented in capitals). The remaining 20 were assigned to a pseudoword condition (e.g., “seed” – DRAGE, “seat” – DRAGE). In addition, 80 of the recorded filler words were paired with pseudoword targets (e.g., “gin” – DORSE), and the remaining 40 were paired with unrelated word targets (e.g., “ring” – MYTH). Six lists were constructed in which the assignment of all critical words to the related, unrelated, and pseudoword conditions was counterbalanced and which were otherwise identical. All lists thus consisted of 240 trials in which half the targets were pseudowords, and across lists the items from the four conditions of interest were equally likely to occur in a related, unrelated, or pseudoword pair.

Stimuli were presented using Presentation software (NeuroBehavioral Systems Inc.) running on a laptop computer. Audio stimuli were delivered via Sennheiser HD280 headphones; visual prompts were shown for 1.4 s in white Helvetica font on black background in the center of the computer screen. Responses were made on a custom response box with two buttons labeled “yes” and “no.” Half of the participants made “yes” responses with their dominant hand. The inter-onset interval in the lexical-decision task was 2.8 s. In the priming task, targets were presented immediately at the offset of the prime and the inter-trial interval was 1.4 s. Reaction times (RTs) in both tasks were measured from the offset of the auditory stimulus. Trials were either ended by a button press, or timed out 1.5 s after target onset.

### Results and discussion

**Figure 1:**
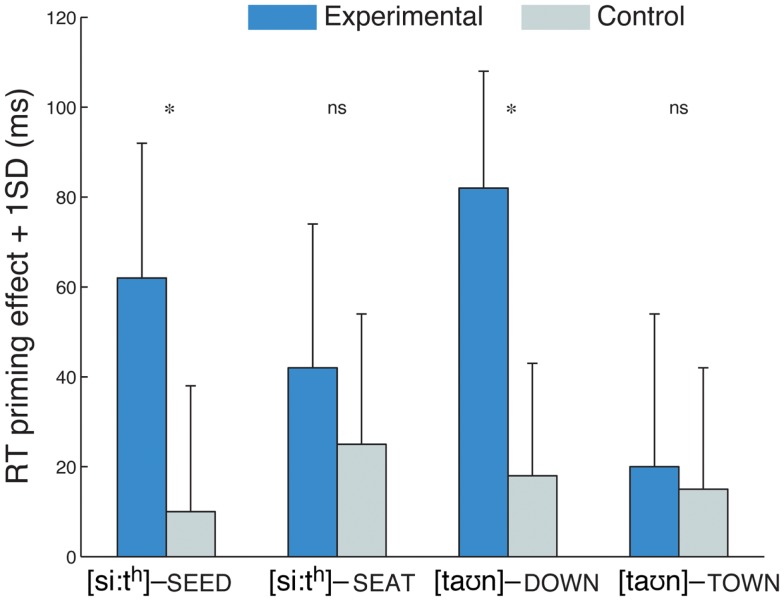
**Priming effects (reaction times in the unrelated minus the related prime type) are shown for each of the four word types, for experimental and control groups, in Experiment 1**. The group differences for the /d/-final and /d/-initial word types illustrate learning for word-final position and generalization to word-initial position, respectively. Starred differences denote a significant interaction of group and prime type (related vs. unrelated).

Analysis of the responses in the lexical-decision task showed that on average, on 78% of the trials in which a finally devoiced item occurred, listeners in the experimental group responded by pressing the “yes” button, indicating that those items were largely judged to be real words. For the priming task, RTs from trials of interest with correct responses (always “yes”; mean error rate: 3%) were analyzed in a mixed ANOVA with the between-subjects factor group (experimental vs. control) and within-subjects factors prime type (related vs. unrelated) and word type (/d/-final, /d/-initial, /t/-final, and /t/-initial). RTs were analyzed separately in a subject analysis (*F*1) and an item analysis (*F*2). Priming effects (unrelated − related RTs) are shown in Figure 1 (see Table A1 in Appendix for mean RTs). The three-way interaction between group, prime type, and word type was significant (*F*1_(3,165)_ = 2.67, *p* < 0.05; *F*2_(3,277)_ = 2.64, *p* < 0.05) and was followed up by four ANOVAs with the factors group and prime type for each word type, as potential effects of exposure would be revealed as an interaction of group and prime type. This interaction was significant for the /d/-final (*F*1_(1,22)_ = 6.48, *p* < 0.05; *F*2_(1,38)_ = 4.43, *p* < 0.05) and /d/-initial (*F*1_(1,22)_ = 9.62, *p* < 0.01; *F*2_(1,38)_ = 5.86, *p* < 0.05) words types, reflecting larger priming effects in the experimental group than in the control group on pairs such as [si:t^h^] – SEED and [ta℧n] – DOWN, but not significant for the /t/-final and /t/-initial word types, that is pairs such as [si:t^h^] – SEAT and [ta℧n] – TOWN (*F*s < 1). *Post hoc* one-tailed *t*-tests for the significant interactions showed that priming effects were significant in the experimental group for /d/-final (*t*1_(1,11)_ = −3.99, *p* < 0.005; *t*2_(1,19)_ = −3.45, *p* < 0.005) and /d/-initial items (*t*1_(1,11)_ = −4.80, *p* < 0.001; *t*2_(1,19)_ = −4.93, *p* < 0.001), but not in the control group (*p*s > 0.05). An analogous three-way ANOVA carried out on error rates did not reveal a significant three-way interaction of group, prime type, and word type, and was thus not followed up further (*F*s < 1).

The results of Experiment 1 indicate that native English speakers could adjust to word-final devoicing in Dutch-accented English speech after having been exposed to 32 devoiced /d/-final words. Participants in the experimental group were faster to decide that a visual target such as SEED is a word when it was preceded by a devoiced production of “seed” than they were when it was preceded by an unrelated item. This priming effect suggests that those participants had learned that the devoiced word-final /d/ in “seed” is an acceptable production for that phoneme category for that speaker, and that the devoiced prime was thus sufficient for activating the intended lexical item and facilitating recognition of the target. In contrast, for the control group, which did not have an opportunity to learn about this aspect of the speaker’s accent, the devoiced /d/ was not a good match for the category; hence no such priming effect occurred for those participants. A likely source of information to drive this learning effect is lexical knowledge (Norris et al., 2003): because the items in which the devoiced stop occurred in the exposure phase were three to four syllables in length, and because those items did not form minimal pairs with voiceless word-final stops, there was overwhelming lexical evidence for the experimental group that the devoicing was an unusual pronunciation, which could override the mismatch at the acoustic-phonetic level. The learning in Experiment 1 was not restricted to word-final items, as the experimental group showed a priming effect for word pairs in which a voiceless alveolar stop consonant in initial position (such as in “town”) produced priming in targets with a voiced initial stop consonant (such as DOWN).

However, neither group of listeners heard the speaker produce canonically voiced word-initial stops in the exposure phase (which is no problem for Dutch learners of English because Dutch distinguishes voicing in onset position too). Previous research on perceptual learning has shown that learning to adjust to an unusual sound may be blocked when there is evidence that the speaker can produce the sound in question correctly (Kraljic et al., 2008; Kraljic and Samuel, 2011). The finding in Experiment 1 raises the question whether a similar blocking process affects the transfer of learning, that is, whether generalization of learning to word-initial position would also occur if canonically voiced, word-initial stops were included in the exposure phase. This question was addressed in Experiment 2.

## Experiment 2

### Materials and methods

#### Participants

Twenty-four undergraduate students enrolled at the University of Dundee participated in exchange for course credit. All participants were native speakers of English, did not speak Dutch, and reported no hearing-related disorders. Participants gave informed consent before taking part in the study. None of the participants had taken part in Experiment 1.

#### Speech materials

Stimulus materials in Experiment 2 differed from Experiment 1 only in that 32 words with a voiced word-initial alveolar stop consonant (e.g., “delivery”) replaced half of the 64 filler words.

#### Design and procedure

Design and Procedure were identical to Experiment 1, except for the substitution of the 32 filler words in the lists of both the experimental and the control group.

### Results and discussion

**Figure 2:**
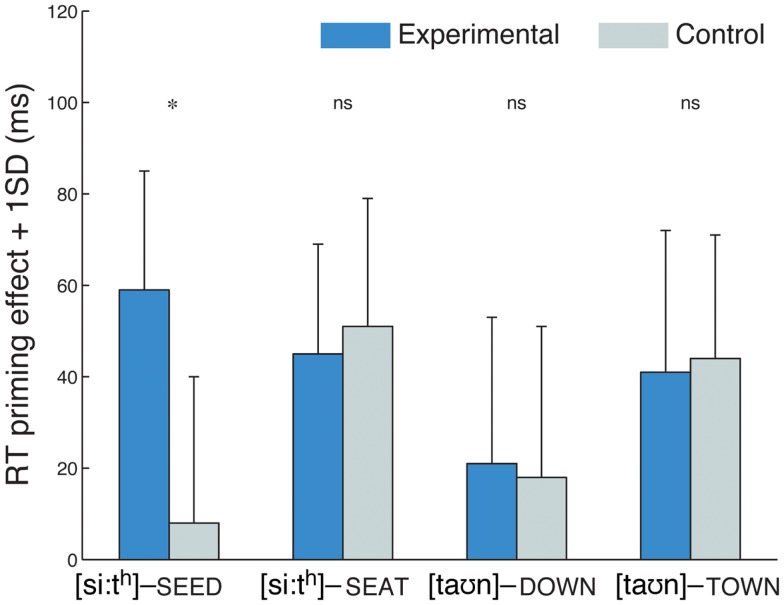
**Priming effects in Experiment 2, showing a group difference for the /d/-final words type but no other word type**. Starred differences denote a significant interaction of group and prime type (related vs. unrelated).

Similarly to Experiment 1, the experimental group classified on average 81% of the word-finally devoiced training items as words in the lexical-decision task. The RTs of the priming task were analyzed as in Experiment 1 in a three-way ANOVA. There was again a significant three-way interaction of group, prime type, and word type (*F*1_(3,165)_ = 2.67, *p* < 0.05; *F*2_(3,277)_ = 2.73, *p* < 0.05) which was followed up by four two-way ANOVA testing a group × prime type interaction for each word type. This interaction was significant only in the case of the word-final word type (*F*1_(1,22)_ = 6.33, *p* < 0.05; *F*2_(1,38)_ = 4.97, *p* < 0.05): as in Experiment 1, participants in the experimental group showed larger priming effects than the control group for word pairs with a devoiced word-final prime and a voiced target (such as [si:t^h^] – SEED; see Figure 2). *Post hoc* tests showed significant priming effects in the experimental group for this word type (*t*1_(1,11)_ = −4.74, *p* < 0.001; *t*2_(1,19)_ = −3.44, *p* < 0.005) but not in the control group (*p*s > 0.05). No significant interaction of group and prime type was observed for any of the three other word types (*F*s < 1).

The results of Experiment 2 thus showed again a learning effect for word-finally devoiced items in the experimental group. Unlike in Experiment 1, however, there was no evidence that this learning generalized to word-initial position when the exposure condition included instances of canonically voiced word-initial stops, as would likely be the case in a more natural listening situation. These results suggest that the composition of linguistic information that is available during exposure to a novel accent can have an effect on aspects of perceptual learning, at least in a tightly controlled laboratory setting. Experiment 3 investigated whether, in addition to linguistic information, paralinguistic information such as speaker characteristics, can have an effect on learning. Previous studies have shown that the way in which listeners processes phonemic information is sensitive to the perceived accent of a speaker (Hay et al., 2006a,b; Hay and Drager, 2010). Furthermore, foreign accents typically contain a range of unusual pronunciations toward which listeners may become more tolerant than they would be if they were produced by a native speaker. Whereas the speaker in Experiments 1 and 2 had a global foreign accent of which the word-final devoicing was only one aspect, Experiment 3 was conducted with recordings from a native English speaker who was instructed to produce word-final devoicing.

## Experiment 3

### Materials and methods

#### Participants

Twenty-four undergraduate students enrolled at the University of Dundee participated in exchange for course credit. All participants were native speakers of English, did not speak Dutch, and reported no hearing-related disorders. Participants gave informed consent before taking part in the study. None of the participants had taken part in Experiments 1 and 2.

#### Speech materials

The materials from Experiment 1, which did not include any /d/-initial words, were recorded by a female, native English speaker with a standard southern British English (SSBE) accent. The recorded materials were identical to those used in Experiment 1, but in addition the speaker was instructed to produce a set of the 32 /d/-final training items in which the word-final stop was intentionally devoiced, reading from a list of adjusted orthographic transcriptions (e.g., “overloat”). The speaker’s productions of critical test items with word-final stops are compared to those of the Dutch speaker in the “Acoustical analysis” section below.

#### Design and procedure

Design and Procedure were identical to Experiment 1, but all stimulus materials were now replaced by the versions recorded by the native English speaker. Critically, the /d/-final training items in the exposure phase, which had been naturally devoiced by the Dutch speaker in Experiments 1 and 2, were now replaced by intentionally mispronounced, devoiced versions by the English speaker.

### Results and discussion

**Figure 3:**
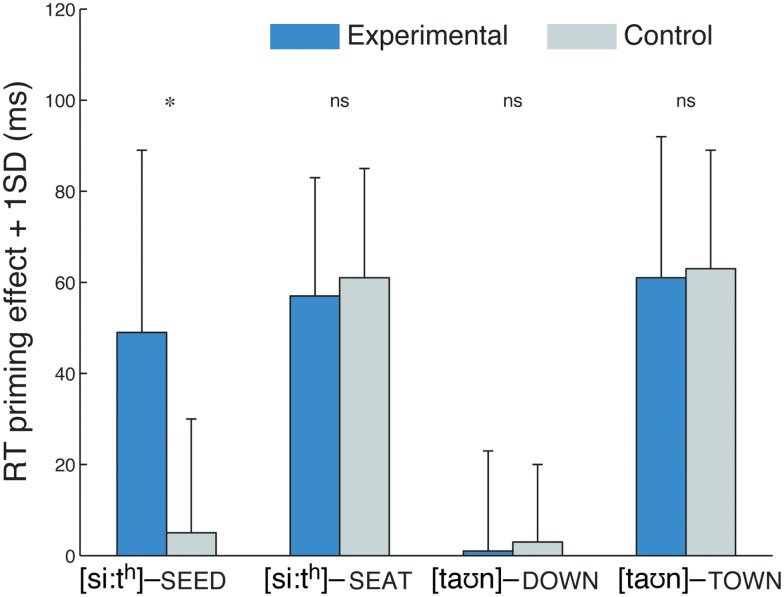
**Priming effects in Experiment 3**. As in Experiment 2, there was a group difference for /d/-final words type but no other word type. Starred differences denote a significant interaction of group and prime type (related vs. unrelated).

Analysis of the lexical-decision data revealed that 71% of the devoiced /d/-final training items were labeled as words by participants in the experimental group. The priming data were analyzed analogous to Experiments 1 and 2. The three-way interaction of group, prime type, and word type was significant (*F*1_(3,165)_ = 2.69, *p* < 0.05; *F*2_(3,277)_ = 3.01, *p* < 0.05). The two-way ANOVA for the four word types revealed a significant interaction for the /d/-final word type ([si:t^h^] – SEED; *F*1_(1,22)_ = 5.1, *p* < 0.05; *F*2_(1,38)_ = 5.43, *p* < 0.05) but not for the other three word types (*F*s < 1), indicating a significantly larger priming effect for the experimental group than for the control group (see Figure 3). *Post hoc* tests confirmed that there was significant priming in the experimental group (*t*1_(1,11)_ = −4.43, *p* < 0.005; *t*2_(1,19)_ = −4.80, *p* < 0.001) but not in the control group (*p*s > 0.05). Experiment 3 showed, in line with the results of Experiments 1 and 2, that listeners readily learned to adjust to word-final devoicing of alveolar stops. Unlike in Experiment 1, there was no evidence for a learning effect in word-initial position, suggesting that the change to a native English speaker affected the readiness of listeners to generalize learning from word-final to word-initial position.

## Acoustical Analysis

**Figure 4:**
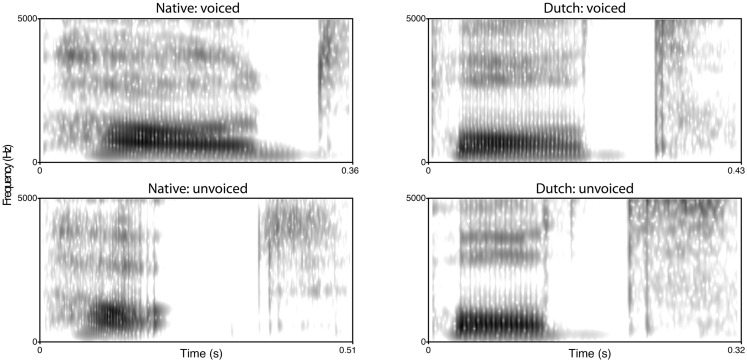
**Spectrograms of the words “pod” and “pot” as produced by the native English (left panels) and the Dutch speaker (right panels)**.

**Figure 5:**
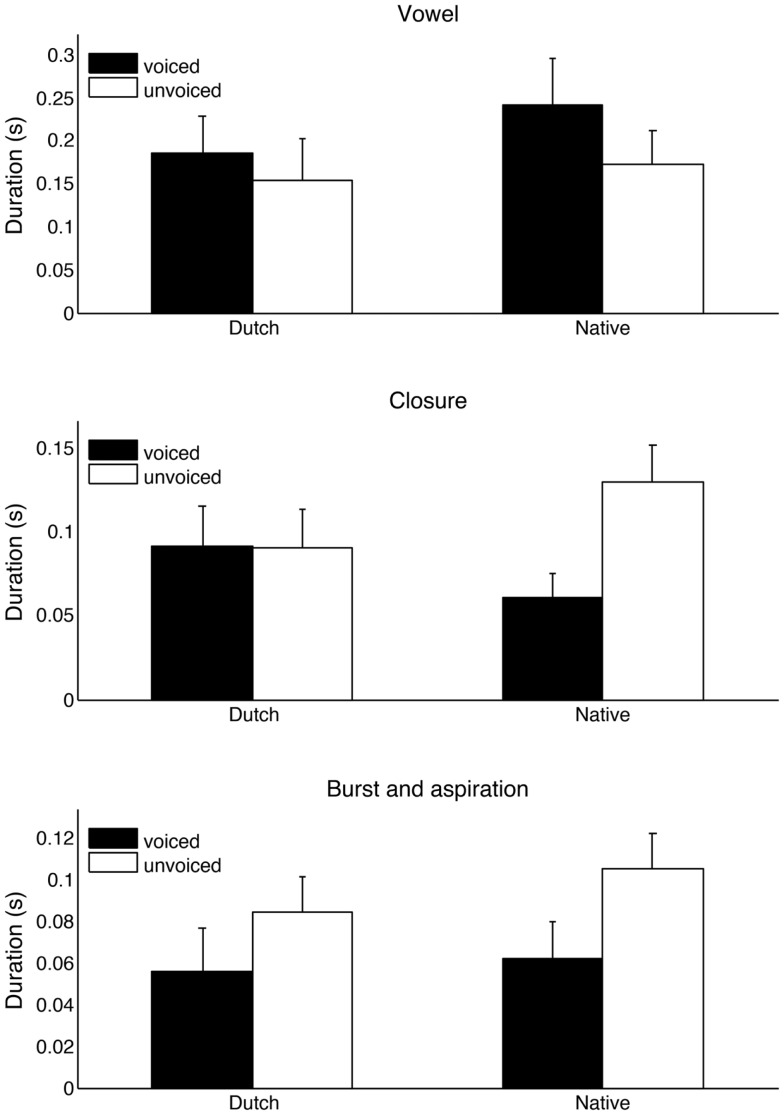
**Duration measurements for both speakers across all minimal pairs with word-final stops in the experiments, shown separately for the voiced and unvoiced members of a pair (e.g., “pod” vs. “pot”)**. Duration distinctions made by the native speaker were reduced or absent for the Dutch speaker in the case of preceding vowel and closure, but mostly preserved for burst and aspiration. Error bars represent standard deviations.

In order to gain a better understanding of the phonetic differences between the native speaker in Experiment 3 and the Dutch speaker in Experiments 1 and 2 with respect to word-final stop production, the critical items with word-final stops were analyzed acoustically using Praat (Boersma and Weenink, n.d.). For this comparison, both items of a critical minimal pair were used, for example, speakers were intending to produce “seed” and “seat”. Figure 4 shows spectrograms of the words “pod” and “pot” produced by the two speakers. In addition to the presence and absence of vocal fold vibration during the closure, there are timing differences affecting the vowel, closure, and burst and aspiration between the words in the case of the native speaker. These differences are attenuated or absent in the case of the Dutch speaker. To quantify those duration based differences, vowel, closure, and burst and aspiration duration was measured for all minimal pairs in the stimulus set. The results (see Figure 5) show that, in general, some phonetic distinctions made by the British speaker are reduced or neutralized by the Dutch speaker (vocal fold vibration, vowel, and closure duration) while others are largely preserved (burst and aspiration duration). Together these results illustrate that the word-final devoicing by the Dutch speaker affects more than one acoustic dimension and that it affects relevant dimensions differently. The incomplete nature of the devoicing is consistent with previous findings for Dutch (e.g., Ernestus and Baayen, 2007) and Dutch-accented English (Warner et al., 2004), and suggests a transfer of this aspect of native-language phonology to the second language.

## General Discussion

Three experiments investigated how native English speakers adjust to word-final devoicing of an alveolar stop consonant. Experiments 1 and 2 demonstrated that listeners readily learned to interpret devoiced word-final consonants in the speech of a native Dutch talker after relatively brief exposure. They learned in a way that ensured unhindered lexical access when tested on a set of words they had not heard during exposure but which were devoiced in the same manner. This learning generalized to test items with word-initial devoicing, indicating that the re-tuning of a native phoneme category in response to a foreign accent may be, at least initially, relatively broad. However, generalization to word-initial position was only observed when the speaker had a global foreign accent and when the speech that was heard during exposure was also highly restricted, not containing any naturally voiced stops. These results add to an emerging body of research that has aimed to describe under which conditions perceptual learning in response to unusual speech occurs, and to what extent the subsequent application of the learning is constrained (Bradlow and Bent, 2008; e.g., Nygaard et al., 1994; Eisner and McQueen, 2005, 2006; Kraljic and Samuel, 2005, 2006; Dahan and Mead, 2010; Sjerps and McQueen, 2010; Jesse and McQueen, 2011).

The adjustment in processing of the devoiced stop was most likely driven by the lexical context provided by the training items: as in studies that have explicitly investigated lexically driven perceptual learning of an ambiguous sound (Norris et al., 2003), including stop voicing contrasts (Kraljic and Samuel, 2006), the three to four syllable context preceding the devoiced sound was sufficient to change the processing of the devoiced /d/category after repeated exposure. In contrast to studies modeled on the paradigm by Norris and colleagues, the devoiced sounds here were a natural characteristic of the Dutch-accented English (Warner et al., 2004) and not obtained through a specific instruction to the speaker or by means of artificial signal manipulation. Perhaps unsurprisingly, lexically driven learning appears to be just as effective for dealing with variation in natural speech as with variation created in “laboratory” accents. It is possible, however, that there were bottom-up factors driving the adjustment as well. As shown by the acoustical analyses, the neutralization of the phonetic cues signaling the voicing distinction in English was incomplete for some dimensions. Listeners may have exploited contingencies between these cues, and inferred the identity of the intended category from any residual cues (Idemaru and Holt, 2011). Because in naturally accented speech, the realization of critical phonetic cues may vary in an uncontrolled fashion, it is difficult to pinpoint the locus of perceptual learning. A lexically driven learning mechanism is likely because of the experimental set-up, but other sources might have contributed. Previous research has suggested that this type of learning takes place at a sublexical level, affecting the mapping of acoustic features to phonemic categories, and may then generalize across the lexicon (Cutler et al., 2010). Perceptual learning effects measured through priming at the lexical level do not necessarily have a one-to-one mapping at sublexical levels, but a sublexical locus of the adjustment is consistent with previous studies that have shown lexically driven learning both with tasks tapping both lexical and sublexical levels.

The differences in speakers and exposure conditions between the experiments also appear to have affected the behavior of the control groups. In Experiment 1, control listeners showed generally weak priming effects, even in word-initial conditions where the intended prime and target were identical. In contrast, control listeners showed strong identity priming effects in Experiment 3, as would be expected. In Experiment 2, identity priming effects were intermediate, somewhat in between Experiments 1 and 3, and comparable to the experimental group. The differences between Experiments 1 and 3 may be explained by sociophonetic differences between the speakers, marked both by differences in the production of the critical sounds, and by the context in which those sounds occur. Whereas in Experiment 3 the control listeners could match the speaker’s native productions relatively easily to their native categories, this would have been less straightforward for those in Experiment 1: they had no opportunity for learning about the Dutch speakers’ stop productions in the exposure phase, and the critical items in the test phase provided few clues because they were minimal pairs. In addition, word-initial voiceless stops in Dutch and Dutch-accented English tend to be unaspirated, which may make them more similar to English voiced stops, and contribute to the confusability of the speaker’s stops in general. Regarding the differences in identity priming between Experiments 1 and 2, we suggest that Experiment 2 provided more opportunity to learn about the Dutch-accented stops because, unlike Experiment 1, both groups heard normally voiced, word-initial stops during the exposure phase. This provided a perceptual anchor for both groups of listeners, and may have helped both to some extent to cope with the stops in the test phase, in particular with the word-initial ones.

A key question addressed by these experiments is whether a perceptual adjustment to a feature of a global accent which is limited to occurring in a specific phonological context will later only be applied when it occurs in that same context, or whether it will generalize freely to any instances of the feature. Stop consonant devoicing in Dutch-accented English was chosen for this study because this accent feature is naturally restricted to word-final position. Tests for generalization produced a mixed result across manipulations. Generalization to word-initial, alveolar stop consonants was found in Experiment 1, where listeners had not heard any canonically voiced instances of stop consonants in any position. Perceptual learning was not found to generalize in Experiments 2 and 3, however, which only differed from Experiment 1 in that listeners in the experimental group also heard instances of voiced stops word-initially, or in that the speaker did not have a global foreign accent, respectively. Previous research has suggested that the generalizability of perceptual learning in speech may vary depending on a variety of factors, such as acoustic relatedness and the type of adjustment being made. Jesse and McQueen (2011) found full transfer of learning from word-final to initial position in a study using ambiguous fricatives (but little evidence that learning occurred in initial position), whereas position-sensitive generalization of learning was found by Dahan and Mead (2010) in a study using spectrally degraded speech. Perceptual learning of a stop voicing contrast may be more resistant to generalization across word positions because stops tend to be acoustically variable and less salient in word-final position than word-initially (Redford and Diehl, 1999). Unlike in the study by Jesse and McQueen, which used the same spliced token of a fricative during learning and test, here each realization of a stop consonants was unique, and thus acoustically more variable, which may have contributed to learning being more resistant to generalization (Kraljic and Samuel, 2005). The negative result in Experiment 3 suggests that the readiness of the perceptual system to generalize learning is also influenced by perceived characteristics of the speaker’s accent. Perceptual learning, including transfer of learning, can be influenced by paralinguistic aspects of the talker (Nygaard et al., 1994; Eisner and McQueen, 2005; Kraljic and Samuel, 2007; Bradlow and Bent, 2008) as well as context (Kraljic et al., 2008). Here, the speaker’s familiar SSBE accent may have prevented overgeneralization across word position because listeners already have relatively stable representations for word-initial stops in that accent. Listeners in Experiment 1, in contrast, may have overgeneralized to word-initial position because they did not have an opportunity to learn about the speaker’s word-initial stop production. At the same time, a reduced general sensitivity to pronunciation errors caused by the global accent, as recently shown for syntactic errors (Hanulíková et al., 2012), may have further contributed to the increased generalizability. Together with the results of previous studies, these findings suggest that generalization of lexically driven perceptual learning is affected by a combination of acoustic, phonological, and sociophonetic factors.

## Conclusion

Native English listeners adjusted readily to word-final devoicing of stops, both in Dutch-accented and in native-accented English. The learning mechanism driving this adjustment appears to generalize relatively broadly in foreign-accented speech after only limited exposure. After sufficient exposure, word position can restrict this readiness to apply the learning. Long-term representations of native accents and the perceived characteristics of the speaker can further modulate when a pre-lexical re-tuning of a phoneme category is applied.

## Conflict of Interest Statement

The authors declare that the research was conducted in the absence of any commercial or financial relationships that could be construed as a potential conflict of interest.

## Appendix

**Table A1:** **Average reaction times in milliseconds and error rates (in brackets)**.

	/d/-Final		/t/-Final		/d/-Initial		/t/-Initial	
	Related	Unrelated	Related	Unrelated	Related	Unrelated	Related	Unrelated
**EXPERIMENT 1**								
Experimental	562 (2)	625 (1)	504 (2)	546 (3)	549 (3)	629 (3)	492 (1)	516 (2)
Control	603 (3)	620 (4)	498 (1)	526 (2)	528 (2)	548 (4)	502 (3)	521 (4)
**EXPERIMENT 2**								
Experimental	490 (5)	549 (3)	515 (1)	562 (2)	577 (3)	599 (4)	483 (4)	526 (3)
Control	597 (3)	606 (1)	509 (2)	563 (4)	562 (1)	581 (5)	471 (1)	518 (5)
**EXPERIMENT 3**								
Experimental	489 (2)	541 (3)	512 (4)	570 (6)	559 (1)	561 (5)	481 (1)	545 (4)
Control	589 (1)	598 (2)	503 (4)	566 (3)	549 (3)	552 (2)	475 (3)	541 (3)
